# Supplementary material for: AffyMAPSDetector: a software tool to characterize Affymetrix GeneChip™ expression arrays with respect to SNPs
Source: BMC Bioinformatics. 2007 Jul 30;8:276. doi: 10.1186/1471-2105-8-276 (PMC1959249; doi:10.1186/1471-2105-8-276)
Supplement: Additional file 13 — AffyMAPSDetector v1 source code. AffyMAPSDetector source code. [file 1471-2105-8-276-S13.zip › AffyMAPSDetector_v1_src/doc/affychip/snpdetector/util/PmMmHeatmapViewerModel.RowComparatorForDataSorting.html]

PmMmHeatmapViewerModel.RowComparatorForDataSorting


|  |  |  |  |
| --- | --- | --- | --- |
| **Overview** \| **Package** \| **Class** \| **Use** \| **Tree** \| **Deprecated** \| **Index** \| **Help** | | |  |
| **PREV CLASS**   **NEXT CLASS** | **FRAMES**    **NO FRAMES**     **All Classes** |  |  |
| SUMMARY: NESTED \| FIELD \| CONSTR \| METHOD | DETAIL: FIELD \| CONSTR \| METHOD |  |  |


---


## affychip.snpdetector.util Class PmMmHeatmapViewerModel.RowComparatorForDataSorting

```
java.lang.Object
  affychip.snpdetector.util.PmMmHeatmapViewerModel.RowComparatorForDataSorting
```

**All Implemented Interfaces:**: java.util.Comparator

**Enclosing class:**: PmMmHeatmapViewerModel

---

public static class **PmMmHeatmapViewerModel.RowComparatorForDataSorting** extends java.lang.Object implements java.util.Comparator

---

**Constructor Summary**

`PmMmHeatmapViewerModel.RowComparatorForDataSorting()`


|  |  |
| --- | --- |
| **Method Summary** | |
| `int` | `compare(java.lang.Object a, java.lang.Object b)` |

**Methods inherited from class java.lang.Object**

`clone, equals, finalize, getClass, hashCode, notify, notifyAll, toString, wait, wait, wait`

**Methods inherited from interface java.util.Comparator**

`equals`

**Constructor Detail**

### PmMmHeatmapViewerModel.RowComparatorForDataSorting

```
public PmMmHeatmapViewerModel.RowComparatorForDataSorting()
```


**Method Detail**

### compare

```
public int compare(java.lang.Object a,
                   java.lang.Object b)
```

:   **Specified by:**: `compare` in interface `java.util.Comparator`


---


|  |  |  |  |
| --- | --- | --- | --- |
| **Overview** \| **Package** \| **Class** \| **Use** \| **Tree** \| **Deprecated** \| **Index** \| **Help** | | |  |
| **PREV CLASS**   **NEXT CLASS** | **FRAMES**    **NO FRAMES**     **All Classes** |  |  |
| SUMMARY: NESTED \| FIELD \| CONSTR \| METHOD | DETAIL: FIELD \| CONSTR \| METHOD |  |  |


---
